# The published complete mitochondrial genome of *Eptesicus serotinus* is a chimera of *Vespertilio sinensis* and *Hypsugo alaschanicus* (Mammalia: Chiroptera)

**DOI:** 10.1080/23802359.2020.1785349

**Published:** 2020-07-06

**Authors:** George Sangster, Jolanda A. Luksenburg

**Affiliations:** a Department of Bioinformatics and Genetics, Swedish Museum of Natural History, Stockholm, Sweden; b Naturalis Biodiversity Center, Leiden, The Netherlands; c Institute of Environmental Sciences, Leiden University, Leiden, The Netherlands; d Department of Environmental Science and Policy, George Mason University, Fairfax, VA, USA

**Keywords:** Chimera, misidentification, mitochondrial genome, Vespertilionidae

## Abstract

The mitogenome of *Eptesicus serotinus* (Serotine bat) was published in 2013 with GenBank accession number KF111725 and NCBI Reference Sequence number NC_022474. This sequence was placed with *Vespertilio sinensis* (Asian parti-colored bat) in a COI gene tree but with *Hypsugo alashanicus* (Alashanian pipistrelle) in a cytochrome *b* gene tree. Direct comparison of mitogenomes showed that 92.4% of this mitogenome is similar to *Vespertilio sinensis*, 5.9% to *Hypsugo alaschanicus*, and that 1.6% of the mitogenome could not be attributed to either species, or any other species. This mitogenome has been re-used in at least 17 phylogenies. Our findings suggest that mitogenomes are best verified with multiple gene trees, followed by direct comparison of sequences. We conclude that greater vigilance is warranted to ensure that problematic sequences do not enter the scientific record, and are not re-used in subsequent studies.

## Introduction

During the last two decades, complete mitochondrial genomes (hereafter mitogenomes) have begun to shed light on the phylogenetic relationships among bats, both at deep phylogenetic levels and among species (Nikaido et al. 2000; Meganathan et al. 2012; Botero-Castro et al. 2013). One of these, the mitogenome of *Eptesicus serotinus* (Serotine bat; KF111725/NC_022474) was released on GenBank in September 2013 and was accompanied by a descriptive paper which appeared online in July 2013 (Nam et al. 2015). No information was provided on its collection locality, preservation state (specimen or tissue sample only) and collection number (if any). At the time of publication, only few bat mitogenomes had become available and even today this is still the only mitogenome purportedly of this species. As a result, KF111725 or NC_022474 have been re-used in at least 17 phylogenies (Hwang et al. 2016; Locatelli et al. 2016; Monadjem et al. 2016; Qian et al. 2016; Yu et al. 2016; Zhang et al. 2016; Jebb, Foley, Kerth, et al. 2017; Jebb, Floey, Puechmaille, et al. 2017; Kim, Kim, et al. 2017; Kim, Park, et al. 2017; López-Wilchis et al. 2017; Mata et al. 2017; Shi et al. 2017; Platt et al. 2018; Huang et al. 2019; Yue et al. 2019; Tang et al. 2020).

In his PhD dissertation, Botero-Castro (2014) noted that KF111725 is not a mitogenome of *E. serotinus* but represents a misidentified *Vespertilio sinensis* (Asian parti-colored bat). Here we show that this mitogenome is actually a chimera of *V. sinensis* and *Hypsugo alaschanicus* (Alashanian pipistrelle) and indeed does not contain any DNA fragments of *E. serotinus*.

## Materials and methods

We verified the identity of KF111725 using sequences from two protein-coding genes: cytochrome oxidase subunit I (COI, 698 bp) and cytochrome *b* (cyt *b*, 1140 bp). These are the two most commonly used mitochondrial markers in chiropteran systematics. A phylogeny was also constructed using sequences of the 13 protein-coding genes (PCGs) included in the mitogenome but with the data set trimmed by GBLOCKS (Castresana 2000). GBLOCKS eliminates poorly aligned positions and divergent regions, which may not be homologous or may have been saturated by multiple substitutions (Castresana 2000). This resulted in an alignment of 11,328 bp. The MITOS2 web server (Bernt et al. 2013) was used to obtain information on the first and last positions of individual genes. CLUSTALW (as implemented in MEGA7, Kumar et al. 2016) was used to align sequences. Maximum Likelihood phylogenies were obtained using MEGA7. The appropriate substitution model for each data set was selected using the Akaike Information Criterion. In all cases, the GTR + G + I model was selected. Sequence divergence was calculated as uncorrected p-values with complete deletion of nucleotide positions with missing data.

## Results

**Figure 1.**
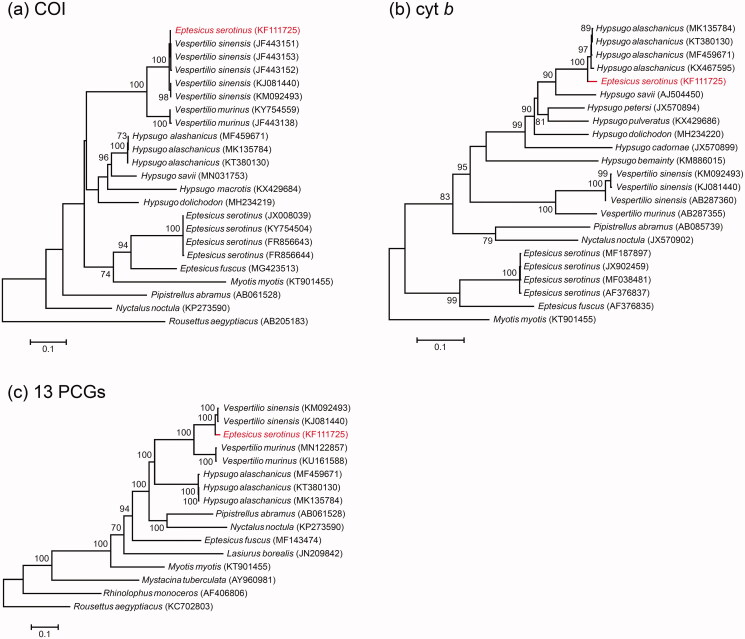
ML phylogenies of vespertilionid bats and selected outgroups based on (a) COI (698 bp), (b) cytochrome *b* (1140 bp) and (c) mitogenomes (13 PCGs, trimmed with GBLOCKS; 11,328 bp). Numbers along branches represent bootstrap support values (>70%) based on 1000 pseudoreplications. Note that KF111725 (*Eptesicus serotinus*) clustered with *Vespertilio sinensis* in the COI gene tree, but with *Hypsugo alashanicus* in the cytochrome *b* gene tree.

In the COI gene tree (Figure 1(a)), KF111725 was part of a strongly-supported clade with five sequences of *V. sinensis*, to which it was closely similar (0.2–0.6% sequence divergence). In contrast, in the cyt *b* gene tree (Figure 1(b)) KF111725 was part of the *Hypsugo* clade, sister to four *H. alashanicus* from which it was 2.5–2.9% divergent. Direct comparison of aligned sequences showed that bp 1–136 of the cyt *b* fragment of KF111725 were a close match with *V. sinensis* (0–0.7% divergence), whereas bp 137–1140 were a close match with four *H. alaschanicus* (0.3–0.9% divergence).

ML analysis of the GBLOCKS-trimmed 13 PCGs placed KF111725 sister to two *V. sinensis* (Figure 1(c)) but differed from those sequences by 2.7–3.0%. Direct comparison of KF111725 with the full mitogenomes of *V. sinensis* (KJ081440, KM092493) and *H. alaschanicus* (MK135784, MF459671, KT380130) showed that 92.4% (bp 1–14,301, 15,308–15,895, 16,151–16,673) of this mitogenome is that of *V. sinensis* and 5.9% (bp 14,308–15,294) is of *H. alaschanicus*. A fragment of 243 bp (bp 15,908–16,150; 1.6% of the mitogenome) could not be attributed to either species, or indeed to any other species.

## Discussion

The placement of KF111725 with *V. sinensis* in the COI gene tree but with *H. alashanicus* in the cytochrome *b* gene tree is *prima facie* evidence that this mitogenome represents a chimera. Direct comparison of KF111725 with sequences of *V. sinensis* and *H. alaschanicus* clearly show that a major part of the mitogenome is nearly identical to that of the former species, and that another part is identical to that of the latter species. This shows that KF111725 is a chimera of *V. sinensis* and *H. alaschanicus*. The laboratory that produced this mitogenome has indeed also produced mitogenomes of *V. sinensis* (Yoon et al. 2016) and *H. alaschanicus* (Kim and Park 2015; Kim et al. 2019). However, because none of these sequences contained any DNA of *E. serotinus*, we suspect that KF111725 was initially misidentified as *V. sinensis*, followed by transfer of a DNA fragment of *H. alaschanicus*, either as template DNA prior to PCR amplification, or as PCR product prior to sequencing, or as a DNA sequence fragment during sequence assembly/editing. In any case, the problematic nature of KF111725 (and NC_022474) shows that this mitogenome should not be used for any evolutionary applications.

To enable efficient detection of misidentified mitogenomes, Botero-Castro et al. (2016) proposed that mitogenome announcements should include (i) a ML gene tree of the most commonly used barcode marker in the group be used to assess the identity of the mitogenome, and (ii) a ML tree of mitogenomes of closely related species, and that (iii) relationships be depicted with phylograms to show branch lengths. Whilst we agree with these recommendations, our study shows that these may not be sufficient for diagnosing chimeras. Whereas both the COI and mitogenome trees clearly show that KF111725 is not a mitogenome of *E. serotinus*, the placement of KF111725 in a clade with *V. sinensis* could also be explained by misidentification. Similarly, if only the cyt *b* gene tree were used to verify the identity of KF111725, its placement close to, but distinct from, sequences of *H. alashanicus* in itself would not be evidence of a chimera and could also be explained by it being a representative of a previously undocumented population of that species. The discovery of novel lineages within a wide-ranging species is a common finding in bat phylogeography (e.g., Larsen et al. 2012; Juste et al. 2013; Tu et al. 2018). Only when either (i) both COI and cyt *b* gene trees are analyzed, or (ii) cyt *b* sequences are directly compared with those of *V. sinensis* and *H. alaschanicus*, would it become evident that KF111725 is not a misidentified sequence. In addition, neither in the two single gene trees nor in the mitogenome tree KF111725 was placed on an unusually long branch, which is probably another factor that made it difficult to detect and diagnose this sequence as a chimera. Finally, the lack of a *bona fide* mitogenome of *E. serotinus*, and the late publication of a mitogenome of another member of the genus (Big brown bat *E. fuscus*; Platt et al. 2018), likely also contributed to the late diagnosis of this mitogenome as a chimera. We recommend that mitogenomes are best verified with multiple gene trees, and that any potentially problematic mitogenomes are directly compared with those of all relevant species or genera.

Assessment of the consequences of re-usage of KF111725 and NC_022474 in other papers is outside the scope of this paper. However, we note that this mitogenome sequence has already been used (i) for phylogeny reconstruction, either as ingroup (e.g. Qian et al. 2016; Yu et al. 2016; Zhang et al. 2016; López-Wilchis et al. 2017) or outgroup (e.g. Platt et al. 2018), (ii) for primer design (Yoon et al. 2016), and (iii) in a reference database for DNA screening of Finnish stone age sediments (Peltola 2019). We conclude that greater vigilance is warranted to ensure that problematic sequences do not enter the scientific record, and that those that remain undetected do not compromise evolutionary inferences.

## Data Availability

The data that support the findings of this study are openly available on GenBank at https://www.ncbi.nlm.nih.gov/nucleotide. Accession numbers are listed in Figure 1.
